# Spatial distribution, influencing factors and innovative development countermeasures of farmer cooperatives in Hunan Province, China

**DOI:** 10.1038/s41598-024-56773-0

**Published:** 2024-05-17

**Authors:** Liu Bin, Tang Chengli, Zhou Guohua, Li Qiuhong, Yi Chun

**Affiliations:** 1 College of Resources and Environmental Sciences, Hunan Normal University, Changsha, 410081 Hunan China; 2 College of Architecture and Urban Planning, Yiyang, 413000 Hunan China; 3 Key Technology of Digital Urban and Rural Spatial Planning Key Laboratory of Hunan Province, Yiyang, 413000 Hunan China; 4 Key Laboratory of Urban Planning Information Technology in Hunan Province, Hunan City University, Yiyang, 413000 Hunan China

**Keywords:** Farmer cooperatives, Demonstration cooperatives, Spatial pattern, Influencing factors, Hunan Province, Environmental economics, Sustainability

## Abstract

China’s rural reform is reliant on farmers' cooperatives as a key organization vehicle. It plays an important role in promoting rural revitalization. Such as, realizing the organic connection between small farmers and agricultural modernization. This study used the nearest neighbor index and kernel density estimation to analyze the spatial distribution and structural characteristics of farmer cooperatives in Hunan Province. It revealed the spatial differentiation law of cooperatives. Also using geographical detectors to figure out the main factors that affect the spatial distribution. The results show that: ① Hunan Farmers Cooperatives show significant spatial agglomeration. The overall spatial distribution shows the spatial characteristics of "one core, one circle, and multiple points". And the spatial distribution pattern of "large agglomeration, finger-shaped radiation distribution". Among them, the distribution of provincial demonstration cooperatives is relatively balanced. ② Cooperatives in six types of industries, such as planting, forestry, and feeding, showed agglomeration distribution. Different industrial cooperatives spread out in different ways, such as in an anti-"L" shape, a dual-core shape, or a one-center area. ③ The 24 influence factors selected from the five dimensions of the natural environment, social economic basis, production basis, output capacity, and facility basis have high, general, and weak influence on cooperatives' spatial distribution. The development and distribution of cooperatives mainly depend on resource endowment, social and economic development level, and market dependence. The spatial distribution heterogeneity of different professional cooperatives is mainly affected by slope, per capita disposable income of rural residents, road density, and other factors. ④ The progression of farmer cooperatives in Hunan Province should prioritize high-quality development, emphasizing the need for innovative approaches and transformative strategies within rural industrial organizations. It is imperative to optimize the spatial distribution of cooperatives, strategically contributing to the establishment of a novel framework for modern agricultural development in Hunan Province. Additionally, there is a critical emphasis on spearheading collaborative initiatives among cooperatives of varied industrial types, aimed at fostering the integrated development of rural primary, secondary, and tertiary industries.

## Introduction

Farmers' cooperatives evolved from farmers' professional cooperatives. In 2007, the "Farmers' Professional Cooperative Law of China" was promulgated and implemented^1^. It defined a farmers' professional cooperative (FPC) as a voluntary, democratic, and cooperative economic organization based on the contracted management of rural households. The members of this organization include providers and users of agricultural production and operation services. Farmers' professional cooperative is an important organization that meets the needs of rural reform in China^2^. It promotes the new collective economy in rural areas and the all-around reorganization of rural space^3^. In 2013, China's No. 1 Central Document for the first time proposed ' farmers ' cooperatives ', which is a further extension and generalization^4^.

Demonstration cooperatives of specialized farmer cooperatives refer to the advanced and typical cooperatives selected by the Ministry of Agriculture and Rural Affairs, the Ministry of Forestry and other departments according to certain evaluation standards^5^. The construction of demonstration cooperatives is an important measure to promote the development of farmers' professional cooperatives. It is also a standard promotion action of farmers' cooperatives under national policies. As of April 2021, the number of farmers ' cooperatives legally registered in China reached 2.259 million^6^. The Ministry of Agriculture and Rural Affairs has established more than 9000 national, provincial, municipal, and county-level model cooperatives. Among them, there are nearly 160,000 demonstration cooperatives at or above the county level^7^. In 2022, according to the documents of the Ministry of Agriculture of China, there will be 200,000 demonstration cooperatives and demonstration family farms at or above the county level^8^. It indicates that farmers ' cooperatives have become an important force to promote rural revitalization in China. Additionally, it realizes the organic connection between small farmers and agricultural modernization^6^.

Researches on farmer cooperatives have accumulated rich work. The studies on farmers ' cooperatives mainly focus on their organization development^6^, democratic management^8^, system evaluation^9^, and so on. The analysis of the performance and efficiency of cooperatives has become the focus^10,11^. Numerous studies have demonstrated the significant impact agricultural cooperatives have on agricultural production, farmer-family income, and green agriculture^12–15^. Other research revealed that professional cooperatives for farmers help participants improve their scale remuneration, the marginal return on land and labor, and technical efficiency^16^. Notably, studies conducted in Vietnam, India and other Asian countries place a significant emphasis on evaluating the influence of farmers 'cooperatives on farmers' income^17,18^. Through an examination of specialized cooperatives for farmers, certain scholars have investigated living conditions and internal risks^19,20^. Additionally, some researchers have analyzed the evolutionary process of the credit rating of the American farmer cooperatives^21^.

In view of the large scale and uniqueness of Chinese farmers ' cooperatives^22^, there are many research contents. The research scale is mainly based on the specific cooperatives at the micro level. Moreover, the regional scale that continues to the city, province and urban agglomeration is gradually increasing^6,23^. Economic cooperatives, mutual fund cooperatives, agricultural land stock cooperatives, digital cooperatives, and other innovative service cooperatives have emerged in recent years, providing finance, land, leasing, and other innovative services^24,25^. Research indicates that farmers' cooperatives have played a significant role in alleviating poverty in rural China^26^.

Considering the existing research content, the research of domestic and foreign farmers 'cooperatives mostly takes the perspective of micro individuals to discuss farmers' cognition of cooperatives and their willingness to join^27^, or from how the development of cooperatives, what efficiency of cooperatives can produce and other aspects of the discussion^28,29^. However, there is a noticeable gap in the analysis of the overall spatial pattern and distribution of cooperatives. Existing research methods tend to favor qualitative analysis over quantitative approaches. Employing spatial analysis methods and the geographical detector model has become instrumental in unveiling spatial differentiation characteristics^30^. The application of geographical spatial analysis methods is gaining prominence in the analysis and research of the spatial distribution and evolution of cooperatives^23,31^.

Based on the previous research results, this paper analyzes the spatial distribution characteristics of different types of farmer cooperatives in Hunan Province. It aims to explore the influence mechanism and innovative development mechanism of farmer cooperatives, holding substantial theoretical and practical significance. This endeavor contributes to enhancing the practical, comprehensive, and theoretical aspects of rural geography research. Additionally, it provides valuable countermeasures and recommendations to foster the differentiation, diversification, and distinctive development of farmer cooperatives in Hunan Province.

## Study area and data sources

### Overview of the study area

Hunan province is located in the middle part of China, in the middle reaches of the Yangtze River, south of Dongting Lake, dense river network, rich in water resources^32^. Superior natural resources also provide favorable basic conditions for the development of agricultural industry. In recent years, under the guidance of the strategy of "three high and four new", Hunan province is vigorously moving towards the development of agricultural modernization. Supporting the development of various rural professional cooperatives is an important measure to promote the process of agricultural modernization in the province^33^.

In recent years, guided by the "three high and four new" strategy, Hunan Province has been actively pursuing agricultural modernization. Supporting the establishment and growth of diverse rural professional cooperatives stands as a pivotal measure to propel the province's agricultural modernization process^34^. The recent development of cooperatives in Hunan Province exhibits the following distinctive characteristics.

**Figure 1:**
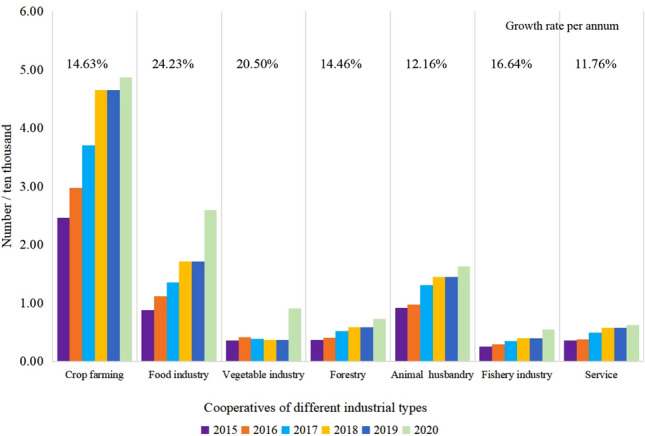
The development of the main industrial types of farmer cooperatives in Hunan Province in recent years.

The overall number of cooperatives has grown rapidly, which has gradually enhanced the ability of rural industrial personnel. According to the number of farmer cooperatives in Hunan province in the past six years published in the Hunan Provincial Rural Statistical Yearbook (2016–2021) (Fig. 1), a total of 93,500 farmer cooperatives have been established in Hunan Province by 2020^35^. Up 51.13 percent from 45,700 in 2016. In 2020, the number of cooperative members was 3.0674 million, the same year as the primary industry employees in Hunan Province. The number of cooperative members reached 36.69% of the total number of employees in rural industries^35^.

**Figure 2:**
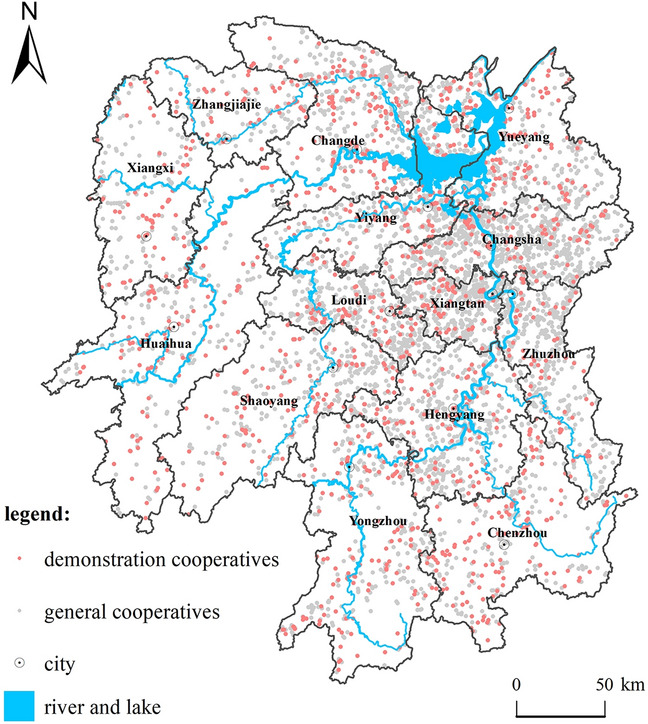
Distribution of research points of farmer cooperatives in Hunan Province. The figure was created by the author, who first cited administrative division vector data, traffic road network data, and other map data from the China Geographic Information Resource Catalog Service System (http://www.webmap.cn) for Geographic Information Resources. Subsequently, the author used ArcGIS 10.6 software (https://desktop.arcgis.com/zh-cn/quick-start-guides/10.6/) to perform spatial analysis and create color-coded maps.

The development of cooperatives of different industrial types is unbalanced. The "Hunan Provincial Rural Statistical Yearbook" counts farmers' cooperatives as five types in planting, forestry, animal husbandry, fishery, and services in agriculture, forestry, animal husbandry, and fishery. In years 2015–2020, According to the analysis of the cooperative composition of five industrial types of planting, forestry, animal husbandry, fishery, agriculture, forestry, animal husbandry and fishery service industry (Fig. 2)^35^, The number of planting cooperatives ranks first, Grain and vegetable cooperatives have developed especially rapidly, The average annual growth rate reached 24.23% and 20.50%, respectively; Second by livestock cooperatives, Growth was steady, The average annual growth rate was 12.16%; Forestry, forestry, animal husbandry and fishery service cooperatives, The average annual growth rate was 14.46% and 11.76% respectively; The smallest number of fishing cooperatives, But the growth rate is faster, The average annual growth rate is 16.64%.

The proportion of demonstration cooperatives is relatively small, and the overall development level of cooperatives needs improvement. Demonstration cooperatives refer to provincial-level and above specialized farmer cooperatives in Hunan Province (referred to as 'demonstration cooperatives'), including those announced by the Ministry of Agriculture and Rural Affairs, the National Development and Reform Commission, and others as national-level demonstration cooperatives, those announced by the Hunan Provincial Forestry Department, the Agriculture and Rural Affairs Department, and others as demonstration cooperatives for farmer forestry, provincial-level demonstration cooperatives for farmer cooperatives, and modern agricultural machinery cooperatives and demonstration cooperatives (collectively referred to as 'provincial-level demonstration cooperatives')".

Since 2012, the national demonstration cooperatives have been evaluated every two years. As of 2020, Hunan Province hosts 574 national farmer professional demonstration cooperatives, comprising 6.29% of the national total (9114).^36^. Additionally, there are 1768 provincial demonstration cooperatives, including 365 specialized farmer forestry cooperatives and 1175 modern agricultural machinery cooperatives, as well as 228 other demonstration cooperatives (Table 1). Notably, the 2342 demonstration cooperatives above the provincial level in Hunan province constitute a relatively small proportion of the overall cooperative count.

**Table 1:** Types and numbers of demonstration societies above the provincial level.

Model society type		Identify the basis	Cooperative type or scope of business	Quantity (individual)
National demonstration society		Measures for Evaluation and Monitoring of National Farmers' Cooperatives	Including agricultural cooperatives, forestry cooperatives, supply and marketing cooperatives	574
Provincial demonstration society	Modern agricultural machinery cooperative demonstration cooperatives	Notice on Printing and Distributing Implementation Plan of Construction Project of Modern Agricultural Machinery Cooperative in Hunan Province in 2018; Notice on Printing and Distributing Implementation Plan of Modern Agricultural Machinery Cooperative Construction Project in 2020–2022 in Hunan Province	Agricultural machinery operation, agricultural machinery maintenance, agricultural machinery distribution and other agricultural machinery services professional cooperatives	228
	Hunan Farmers forestry professional cooperative demonstration society	Measures for Identification and Monitoring of Hunan Farmers' Forestry Professional Cooperatives	The business content includes afforestation, camellia oil, bamboo, flowers and seedlings, underforest planting, underforest breeding, forest tourism and other professional cooperatives involving all aspects of forestry	365
	Provincial demonstration cooperatives of farmer cooperatives	Notice of Hunan Provincial Commission of Agriculture Office on the Clearing and Identification of Farmers' Provincial Demonstration Cooperatives (No.152, 2017); Measures for Establishing Hunan Provincial Provincial Demonstration Cooperatives (Hunan Agricultural Cooperatives No.16, 2020)	Farmers 'cooperatives and farmers' cooperatives' associations in advantageous and characteristic industries such as grain, livestock, poultry, vegetables, tea, oil, Chinese medicinal materials and aquatic products also include agricultural industrialization associations established with leading enterprises and family farms	1175
Total				2342

### Research methods

#### GIS spatial analysis

(1) Nearest neighbor index

The Nearest Neighbor Index (NNI) is a method based on spatial distance. The principle is to randomly select one of the points in the actual data, and compare the average distance (D0) from the nearest point with the expected nearest neighbor distance (DE) in the random distribution mode. The ratio is used to judge the spatial aggregation of the 'point'. The actual nearest neighbor average observation distance formula is as follows^37^:

$$ \overline{{{\text{D}}_{0} }} = \frac{1}{{{\text{n}}_{1} }}\sum\limits_{i = 1}^{n} {d_{i} } $$

In the formula: di is the distance between the ith point and its nearest neighbor; n1 is the number of points. The formula for calculating the expected nearest neighbor average distance is as follows:

$$ D_{E} = \frac{1}{2}\sqrt {n_{2} /A} $$

In the formula: n2 represents the number of farmer cooperatives in each industry type; A is the area of the study area. Then the ratio of the two is calculated and expressed by the nearest neighbor ratio (NNI). When the neighbor ratio is less than 1, the farmer cooperatives are clustered. When the neighbor ratio is close to 1, the farmer cooperatives are randomly distributed. When the neighbor ratio is greater than 1, the farmer cooperatives are evenly distributed. In order to further reflect the deviation between the measured average distance and the expected average distance, this study uses the normal distribution test to obtain the Z score and its confidence level P. The Z score is negative, and the smaller it is, the more the feature distribution tends toward agglomeration. If it is positive and larger, the features significantly lean towards a uniform distribution. If it is situated between the two, the distribution is considered random^37^.

(2) Kernel density estimation

Kernel density estimation (KDE) is a non-parametric method that uses the peak function to fit known sample points by simulating the actual probability curve^38^. KDE can analyze the density of the elements in the surrounding neighbors according to the location and number of the point elements. It reflects the spatial density of a specific element, allowing for the identification of spatial distribution characteristics of the elements^39^. The kernel density method is a non-parametric estimation method, which can use the spatial properties of the data itself to study the distribution characteristics of spatial data, and can avoid errors caused by pre-specifying a specific distribution^39^. The formula for calculating the kernel density is as follows:

$$ {\text{f}}({\text{x}}) = \frac{1}{{{\text{n}} \times {\text{h}}}}\sum\limits_{{{\text{i}} = 1}}^{{\text{n}}} {{\text{k}}\left( {\frac{{{\text{x}} - {\text{x}}_{{\text{i}}} }}{{\text{h}}}} \right)} $$

In the formula: f(x) is the kernel density function; h is the bandwidth; n is the number of known points; k(x) is the kernel function; x-xi is the distance from the estimated point to the known point i.

#### Geographic detector differentiation and factor detection

The Geographic Detector is a statistical method that utilizes spatial similarity to detect the influence of independent variables on dependent variables, revealing the driving forces behind spatial phenomena. Primarily employed to unveil the driving factors of spatial differentiation phenomena^40^. Geodetector q statistic, which can be used to measure spatial heterogeneity, detect explanatory factors, and analyze interactions between variables. It has been widely used in geographic research^40^. This study assumes the cooperative core density y in the area where the cooperatives in Hunan Province are located, and the grid point system composed of 8282 cooperatives in Hunan Province is collected in the cooperative core density map to represent. We assume that there may be a factor that affects the spatial differentiation of cooperatives denoted as A = {A h}, h = 1, 2, …, L. L is the classification number of influencing factors, A h is the different types of influencing factors A, and a type h corresponds to one or more regional subsets of the spatial distribution of cooperatives. The degree of explanation of the influence factor A on the core density y of the cooperative is expressed as follows:

$$ {\text{q}} = 1 - \frac{{\sum\nolimits_{{{\text{h}} = 1}}^{{\text{L}}} {{\text{N}}_{{\text{h}}} \sigma_{{\text{h}}}^{2} } }}{{{\text{N}}\sigma^{2} }} $$

In the formula: q is the explanatory power of the influencing factors of the distribution of cooperatives (core density of cooperatives), and its value range is [0, 1]; L is the number of sub-regions of cooperatives divided by the influencing factors. This paper adopts the equal division method to divide into 3 level; h is a sub-region; N h is the number of cooperatives in a sub-region; N is the number of cooperatives in the entire region; σ2h is the kernel density of cooperatives in a sub-region degree variance; σ2 is the variance of the cooperative kernel density in the entire region.

The larger the q value corresponding to a certain influencing factor, the stronger the explanatory power of the factor on the distribution of cooperatives, the greater the influence, and the weaker the vice versa. In the extreme case, a q value of 0 indicates that the influencing factor has nothing to do with the cooperative distribution. When the value of q is 1, it indicates that the influencing factors completely affect the distribution of cooperatives^37^.

### Data sources and processing

#### Base terrain data

The administrative division vector bundance data and traffic road network data come from the 1:1 million basic geographic databases provided by the China Geographic Information Resource Catalog Service System (http://www.webmap.cn). The terrain data such as elevation and slope are from the 30m resolution digital elevation data provided by Geospatial Data Cloud (http://www.gscloud.cn). The traffic road network data is clipped from the Hunan Province part of the China Basic Geographic Information System (https://www.ngcc.cn/ngcc/).

#### Agricultural cooperative POI data

Point of interest (POI) data is a kind of point-like data that represents real geographic entities and contains spatial and attribute information^41^. Geospatial big data, mainly represented by points of interest (POI), emphasizes the full-time and spatial trajectory records of human activities, which is of great innovative significance for the study of human-land relationships^43^. At present, the research content of POI data mainly focuses on urban areas^43^. However, with the development trend of urban–rural integration and the popularization of rural network infrastructure, it provides conditions for the effective supplement of big data mining and research in rural areas. In view of the huge number of farmer cooperatives under the statistics of the agricultural sector, it is difficult to verify and confirm the number of cooperatives that play an actual effect^44^. The use of POI data can be considered as actual feedback on the existence of cooperatives. In this study, the latitude and longitude picking program code were written in Python 3.8. Then obtain the longitude and latitude of various farmers' professional cooperatives in Hunan Province from the AutoNavi map. Finally, a total of 6328 valid relevant data points were obtained after screening. According to the available list of 2342 demonstration communities, the longitude, and latitude were also obtained based on the address resolution method of Baidu Map API. After screening duplicate points, a total of 1954 demonstration community vector point data were obtained and then visualized with ArcGIS software.

In summary, the research data points of farmers ' cooperatives in Hunan Province are 6328 valid data points obtained by POI and 1954 vector point data of demonstration cooperatives, a total of 8282. Its distribution is shown in Fig. 2.

#### POI data industry type classification

Based on the industrial nature of the farmer cooperatives in Hunan Province, 6328 cooperatives and 1954 demonstration cooperatives are split into 6 categories: planting, forestry, animal husbandry, fishery, agriculture, forestry, animal husbandry, and fishery services, and comprehensive categories. Each of these categories is then broken down even further. There are 9 industrial sub-categories, including grain, fruits, and vegetables, Chinese herbal medicines, flowers and seedlings, tea, tobacco leaves, camellia, comprehensive, and others (Table 2). The number of comprehensive industrial cooperatives is the highest among cooperatives, at 28.69%. Most of these cooperatives are in the comprehensive category of planting and breeding. Agriculture, forestry, animal husbandry, and fishery service cooperatives made up the second largest group, with 23.8%. This group includes agricultural machinery services, plant protection services, soil and fertilizer services, land services, and tourism services. The number of planting, forestry, and animal husbandry cooperatives is relatively equal, accounting for 18.27%, 12.57%, and 13.19%, respectively. Among them, fruit and vegetable planting, flower seedling planting, and grain planting account for a large proportion, and tea and Chinese herbal medicine planting also account for a certain proportion. Additionally, fish, shrimp, turtle and other fishery cooperatives account for 3.40%, and the number is relatively small. Moreover, in terms of the number of demonstration cooperatives of different types of industries, the largest proportion is the planting industry, reaching 29.32%, and the fruit and vegetable demonstration cooperatives in the planting industry account for 20.93%. The number of forestry and agriculture, forestry, animal husbandry and fishery service industry demonstration cooperatives is equal, accounting for 19.70% and 18.94% respectively; comprehensive and animal husbandry demonstration cooperatives account for 15.40% and 12.28% respectively; fishery demonstration cooperatives have the smallest number, accounting for only 3.02%.

**Table 2:** Classification statistics of cooperatives based on industry type.

Industry type		Cooperative		Demonstration cooperative	
		Number (individual)	Proportion (%)	Number (individual)	Proportion (%)
Crop farming	Food stuff	308	4.83	164	8.39
	Fru veg	877	13.74	409	20.93
	Subtotal	1166	18.27	573	29.32
Forestry	Traditional Chinese medicinal materials	91	1.43	61	3.12
	Flowers and trees	430	6.74	103	5.27
	Tea	170	2.66	90	4.61
	Tobacco leaf	15	0.24	25	1.28
	Tea-oil tree	96	1.50	106	5.42
	Subtotal	802	12.57	385	19.70
Animal husbandry		842	13.19	240	12.28
Fishery		217	3.40	59	3.02
Agriculture, forestry, animal husbandry and fishery services		1524	23.88	370	18.94
General works	Comprehensive	1768	27.70	301	15.40
	Other	63	0.99	26	1.33
	Subtotal	1831	28.69	327	16.73
Total		6328	100	1954	100
		8282			

#### Analysis of influencing factors and data sources

Utilizing the nuclear density value of farmer cooperatives in counties and urban areas as the dependent variable, and considering the index distribution characteristics of cooperatives, along with the application and selection criteria of demonstration cooperatives, an index system influencing the spatial distribution of farmer cooperatives is constructed^23,45^. Based on the natural ecological environment, social and economic basis, production basis, output capacity, facilities, five dimensions, mainly selected 125 counties (city, area) GDP, agriculture, animal husbandry and fishery, per capita disposable income, village (house) committee number, rural population, total irrigation area, afforestation area, breeding area, road density of 24 variables as influencing factors (Table 3). The data are from official statistics such as Hunan Statistical Yearbook 2021 and Hunan Rural Statistical Yearbook 2021.

**Table 3:** Variables affecting the spatial distribution of farmer cooperatives in Hunan Province.

Influencing factor	Affectoi	Code	Unit	Affectoi	Code	Unit
Natural ecological environment (A)	Elevation	A1	–	Water area	A3	Hectare
	Slope	A2	–			
Social and economic basis (B)	Village committee	B1	Individual	The GDP of the primary industry	B5	Ten thousand yuan
	rural population	B2	Thousands of people	Total output value of agriculture, forestry, animal husbandry and fishery	B6	Ten thousand yuan
	Per capita disposable income	B3	Ten thousand yuan	Output value of agriculture, forestry, animal husbandry and fishery professional and auxiliary activities	B7	Ten thousand yuan
	Regional GDP	B4	Ten thousand yuan			
Production basis (C)	Area sown with food crops	C1	A thousand hectares	Afforestation area	C5	Hectare
	Total irrigation area	C2	A thousand hectares	Breeding area	C6	A thousand hectares
	Tea garden area	C3	A thousand hectares	Number of pigs and cattle	C7	Ten Thousand head
	Orchard area	C4	A thousand hectares			
Output capacity (D)	Total food crop production	D1	Ton	Total aquatic products	D4	Ton
	Tea production	D2	Ton	Pig and beef production	D5	Ten thousand tons
	Fruit production	D3	Ton			
Facility foundation (E)	Road network density	E1	km/km^2^	Rural electricity consumption	E2	Ten thousand kilowatts per hour

## Results and analysis

### Overall spatial pattern of farmer cooperatives

#### Cooperative spatial agglomeration

The nearest neighbor index of farmer cooperatives and its significance test results show that the nearest neighbor ratios of all cooperatives in the study area are less than 1, showing a significant clustering (Table 4). On the whole, the neighborhood ratio of all cooperative research points is 0.61, and the neighborhood ratio of demonstration cooperatives is 0.42, with strong significant agglomeration. The order of agglomeration degree of different industrial types of cooperatives: forestry > planting > agriculture, forestry, animal husbandry and fishery services > comprehensive > animal husbandry > fishery. Forestry cooperatives nearest neighbor ratio minimum 0.41, indicating that the highest degree of agglomeration. The ratio of planting, agriculture, forestry, animal husbandry and fishery services, comprehensive three types of cooperatives are 0.45, 0.50, 0.58. The nearest neighbor ratios of animal husbandry and fishery cooperatives were 0.59 and 0.69, respectively, and the degree of agglomeration was relatively low.

**Table 4:** Cooperative spatial aggregation analysis.

Cooperative type	P-value price	Z-score price	Near-neighbor ratio (NNI)	Spatial morphology type
All cooperatives	0.00	− 57.94	0.61	Agglomeration
Demonstration cooperative	0.00	− 48.86	0.42	Agglomeration
Crop farming	0.00	− 44.25	0.45	Agglomeration
Forestry	0.00	− 38.81	0.41	Agglomeration
Animal husbandry	0.00	− 26.11	0.59	Agglomeration
Fishery	0.00	− 9.86	0.69	Agglomeration
Agriculture, forestry, animal husbandry and fishery services	0.00	− 41.26	0.50	Agglomeration
General works	0.00	− 37.26	0.58	Agglomeration

#### Overall spatial distribution characteristics

**Figure 3:**
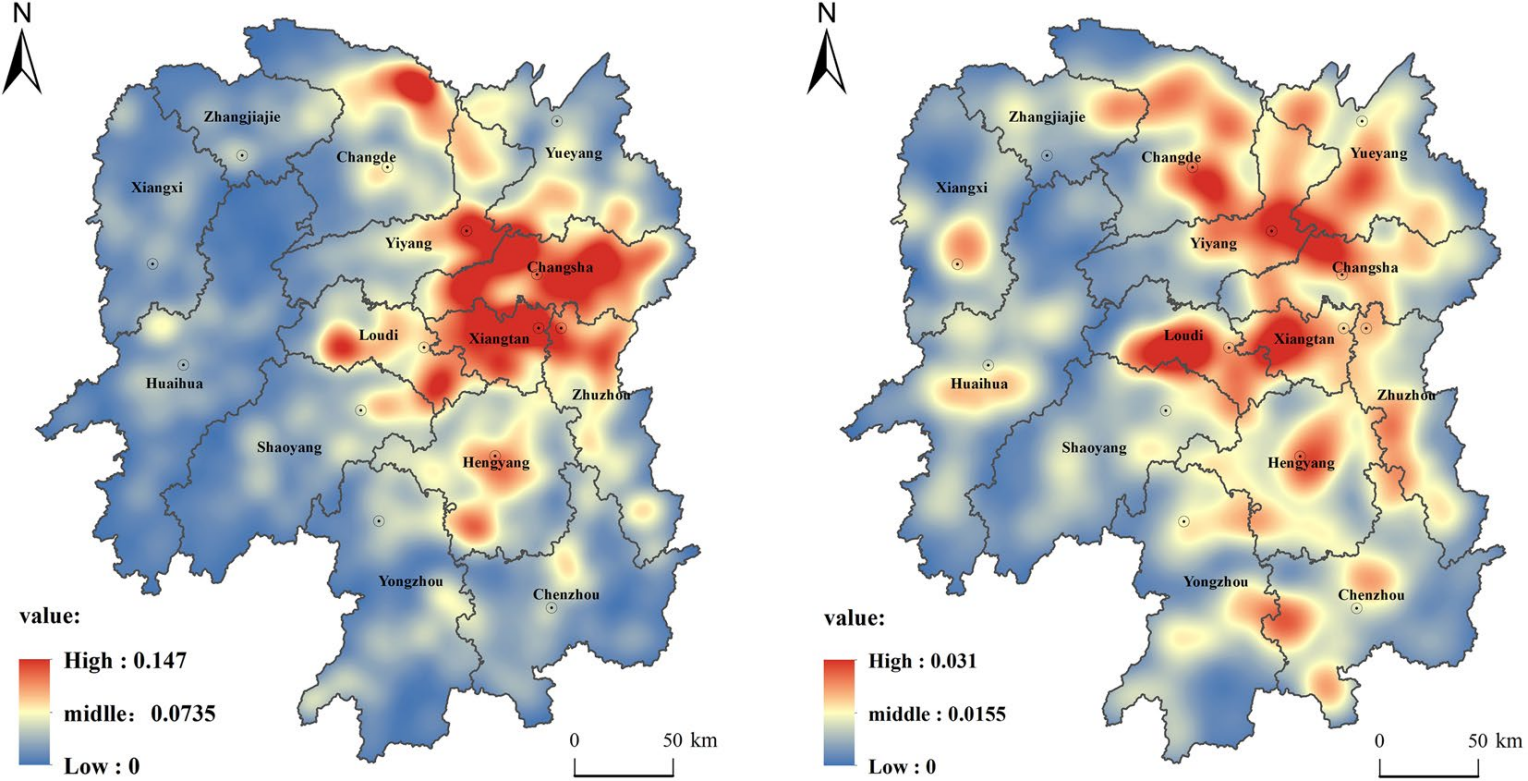
Kernel density analysis of farmer cooperatives in Hunan Province. The Fig. 4 was created by the author, who first used the 30m resolution digital elevation data provided by Geospatial Data Cloud (http://www.gscloud.cn). Subsequently, the author used ArcGIS 10.6 software (https://desktop.arcgis.com/zh-cn/quick-start-guides/10.6/) to perform spatial analysis and create color-coded maps.

A comprehensive analysis of the distribution points of farmer cooperatives in Hunan Province shows the overall spatial characteristics of "one core, one circle, and multiple points" (Fig. 3). The spatial distribution pattern of "large agglomeration and finger-like radiation distribution". The specific spatial characteristics are that Changsha City, Xiangtan City, and Zhuzhou City are the cores, Yiyang City, Yueyang City, and Changde City to the north, Hengyang City to the south, and Loudi City to the west. The Chang-Zhu-Tan urban agglomeration is the main distribution circle of the distribution of farmers' cooperatives in Hunan Province. The northern and central regions of Changde, the central and northern regions of Yiyang, the western and southeastern regions of Loudi, and the central and southern regions of Hengyang constitute the second distribution outer circle. In addition, a number of cooperative distribution areas have been formed in southern Zhangjiajie, central Hunan, central and western Huaihua, northeastern and central Shaoyang, northern and central Yongzhou, and central Chenzhou. Corresponding to the county distribution, except for the key districts (counties, cities) in the Chang-Zhu-Tan urban agglomeration, the distribution of farmer cooperatives in other districts (counties, cities) is in line with the 35 designated in the 2012 "Hunan Provincial Main Function Zone Planning". Corresponding to the county distribution, except for the key districts (counties, cities) in the Chang-Zhu-Tan urban agglomeration, the distribution of farmer cooperatives in other districts (counties, cities) is in line with the 35 designated in the 2012 "Hunan Provincial Main Function Zone Planning". The main producing areas (counties, cities) of agricultural products are relatively consistent, mainly distributed in 15 districts and counties including Dingcheng District, Anxiang County, Hanshou County, Taoyuan County, Yuanjiang City, Nan County, Miluo City, Xiangyin County in the Dongting Lake Plain, and Hengshan County, Hengyang County, Changning City, Qidong County, Shaoyang County, Longhui County and other 20 districts and counties in Hengshao Hilly Area in Hunan Province. In addition, according to the conclusions of the kernel density analysis and the agricultural divisions of Hunan Province, farmers' cooperatives are mainly distributed in the two agricultural areas of the Dongting Lake plain area in northern Hunan and the Changheng hilly basin area in central Hunan. Furthermore, the plain area of Dongting Lake in northern Hunan is dominated by grain, fiber crops and aquatic products. The area of Changheng hilly basin in central Hunan is dominated by grain, pigs, and suburban agricultural areas.

#### Spatial distribution characteristics of demonstration cooperatives

The demonstration cooperatives above the provincial level are significantly concentrated, and a more obvious gathering point has been formed in various cities and states. For example, Changsha, Zhuzhou, and Xiangtan are concentrated in Ningxiang and Wangcheng, Youxian and Chaling, Xiangxiang and Xiangtan; Changde, Yiyang and Yueyang are concentrated in Heshan, Yueyang, and Miluo; Loudi, Hengyang, Chenzhou and Yongzhou are mainly concentrated in Lengshuijiang and Lianyuan, Hengdong and Changning, etc. The provincial-level and above demonstration societies are formed after the independent declaration, audit by the city and county, and audit by experts organized by the Provincial Department of Agriculture and Rural Affairs. They have a strong policy orientation, and the balanced policy implementation needs to be considered as a whole. Therefore, their overall spatial pattern is relatively evenly distributed.

#### Spatial differentiation characteristics of cooperatives based on industrial classification

**Figure 4:**
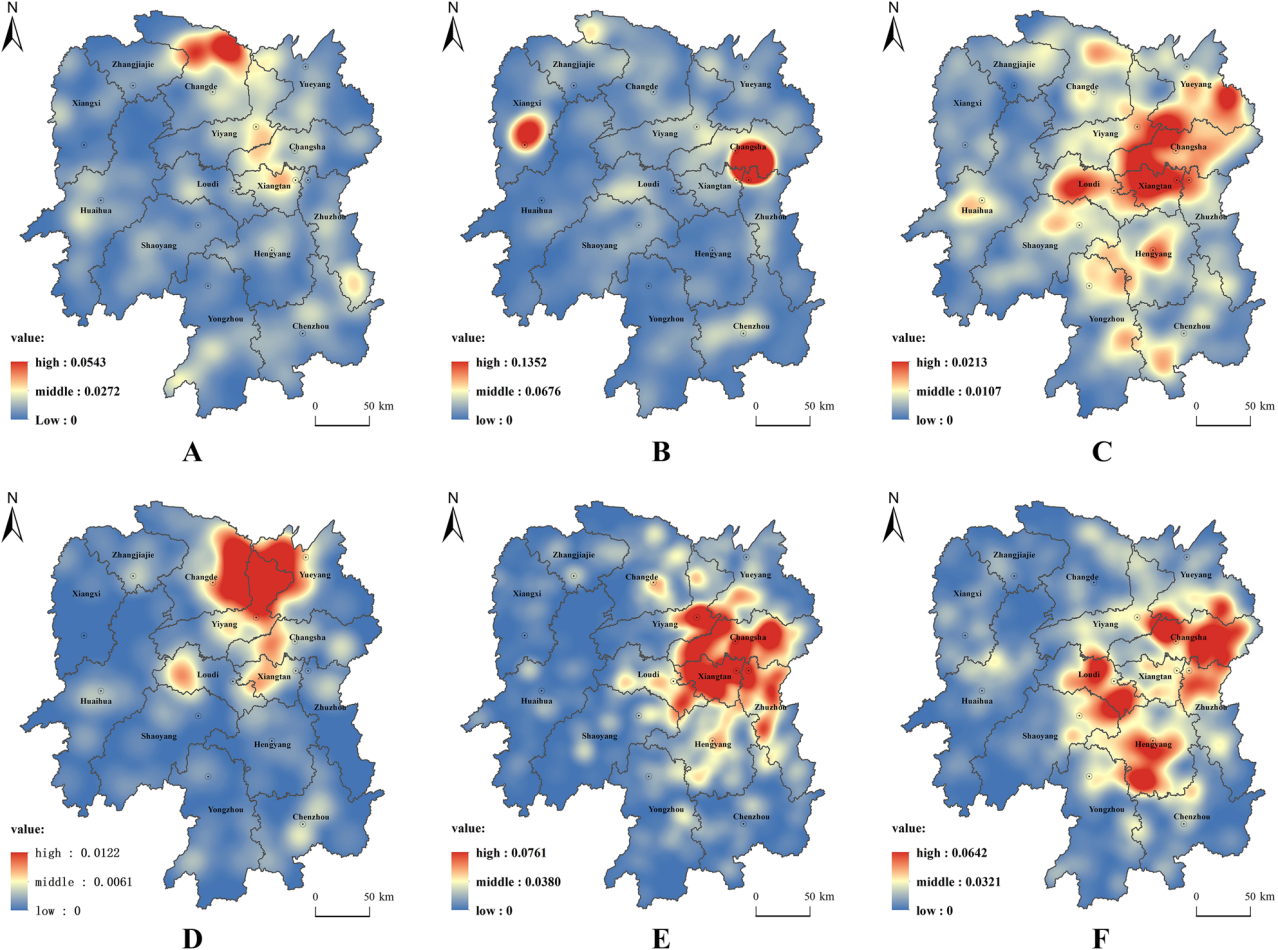
Kernel density map of cooperatives of different industrial types. The Fig. 4 was created by the author, who first used the 30m resolution digital elevation data provided by Geospatial Data Cloud (http://www.gscloud.cn). Subsequently, the author used ArcGIS 10.6 software (https://desktop.arcgis.com/zh-cn/quick-start-guides/10.6/) to perform spatial analysis and create color-coded maps. (**A**) Nuclear density map of planting cooperatives. (**B**) Nuclear density map of forestry cooperatives. (**C**) Nuclear density map of animal husbandry cooperatives. (**D**) Nuclear density map of fishery cooperatives. (**E**) Nuclear density map of agriculture, forestry, animal husbandry, fishery and service industry cooperatives. (**F**) Nuclear density map of comprehensive cooperatives.

Kernel density analysis shows that the spatial distribution characteristics of cooperatives in different industrial types are different (Fig. 4). The anti-"L"-shaped chain space characteristics of planting cooperatives are obvious. Taking the northern part of Changde–central Yiyang–western Changsha–Xiangtan–central and southern Zhuzhou–central Chenzhou–central and southwestern Yongzhou as the main context, an anti-L-shaped chain is formed within the province. In other areas, the central Huaihua, central Hengyang, central and western Hunan, central Shaoyang and western Loudi have slightly obvious point-like spatial distribution. Forestry cooperatives show dual-core spatial characteristics. The Green Heart area, which is the junction of Changsha, Zhuzhou and Xiangtan, and the central part of western Hunan are the two cores. There are some relatively obvious dot-like spatial distributions in the northwest of Changde, the west of Loudi, the central part of Zhuzhou and the west of the central part of Chenzhou. The distribution of animal husbandry cooperatives presents the spatial distribution characteristics of one heart and one area. Western Changsha, Xiangtan and Loudi are the main areas, and central and southern Yueyang form the core distribution area. Central Changde, central Yiyang, the junction of Hengyang and Yongzhou, and the junction of Yongzhou and Chenzhou form a relatively obvious middle distribution zone within the provincial scope. The polar nucleus space characteristics of fishery cooperatives are obvious. The east of Changde, the north of Yiyang and the west of Yueyang in Dongting Lake Eco-Economic Zone are the main core areas. Secondly, there are relatively obvious concentrated distribution areas in central Changsha, central Xiangtan and the west of Loudi. The spatial distribution of cooperatives in agriculture, forestry and fishery services has obvious finger radiation characteristics. Similar to the overall spatial pattern of cooperatives, it takes Changsha and Xiangtan as the core, and the north-central area of Zhuzhou as the core, radiating finger-like to Yiyang, Yueyang and Changde in the north, Hengyang in the south and Loudi in the west. The rest such as Zhangjiajie, Huaihua, Shaoyang, Yongzhou and other regions show a star-like spatial distribution. The spatial distribution of comprehensive cooperatives presents a cluster feature. The central and eastern parts of Changsha and the northern part of Zhuzhou form a group, the central and southern parts of Loudi form a group, and the central and western parts of Hengyang form another group.

### Analysis of factors affecting the spatial distribution of farmers cooperatives

#### Detection of factors affecting the distribution of farmer cooperatives

With the GIS overlay analysis, breakpoint classification, geographic detectors, and other tools, the specific effects of each factor on each professional cooperative were looked at in more detail (Table 5). The impact factors of all cooperative research sites were detected, which showed three obvious levels. ① For the level with high influence, the top three factors of Q value are altitude (0.557), per capita disposable income (0.556), and road network density (0.567) in turn. In addition, the slope (0.514) and regional GDP (0.461) ranked fourth and fifth respectively. ② The influential factors with a general level of influence and a Q value between 0.1 and 0.4 are mostly the number of villages (neighborhood) committees, the rural population, the area where food crops are planted, the total area that is irrigated, the orchard area, the area where trees are planted, the total amount of food crops produced, etc. ③ The level with weak influence, that is, the detection Q value of influencing factors is below 0.1, mainly including water area, the total output value of agriculture, forestry, animal husbandry and fishery, tea garden area, breeding area, tea yield, fruit yield, rural electricity consumption and other influencing factors.

**Table 5:** Detection and analysis of influencing factors of cooperatives.

Code	All types	Crop farming	Forestry	Animal husbandry	Fishery	Service	Comprehensive
A1	0.580	0.388	0.229	0.451	0.660	0.505	0.431
A2	0.535	0.445	0.177	0.467	0.711	0.436	0.421
A3	0.152	0.132	0.141	0.149	0.523	0.138	0.165
B1	0.270	0.140	0.417	0.262	0.119	0.303	0.215
B2	0.271	0.089	0.510	0.256	0.111	0.288	0.305
B3	0.632	0.229	0.707	0.449	0.271	0.657	0.446
B4	0.499	0.143	0.379	0.347	0.121	0.541	0.367
B5	0.229	0.046	0.297	0.183	0.128	0.228	0.224
B6	0.268	0.114	0.318	0.221	0.172	0.263	0.262
B7	0.145	0.109	0.265	0.078	0.188	0.140	0.162
C1	0.317	0.069	0.408	0.278	0.157	0.344	0.281
C2	0.324	0.167	0.412	0.323	0.213	0.294	0.316
C3	0.153	0.081	0.240	0.140	0.201	0.135	0.149
C4	0.429	0.097	0.495	0.358	0.101	0.529	0.245
C5	0.285	0.067	0.539	0.270	0.070	0.392	0.305
C6	0.094	0.272	0.196	0.047	0.510	0.094	0.071
C7	0.274	0.207	0.571	0.214	0.189	0.324	0.220
D1	0.287	0.067	0.371	0.297	0.116	0.231	0.263
D2	0.089	0.090	0.143	0.093	0.246	0.083	0.084
D3	0.236	0.030	0.503	0.252	0.062	0.278	0.182
D4	0.082	0.184	0.121	0.075	0.565	0.052	0.078
D5	0.279	0.170	0.379	0.212	0.154	0.336	0.202
E1	0.582	0.239	0.619	0.430	0.155	0.624	0.423
E2	0.100	0.095	0.034	0.071	0.252	0.073	0.173

#### Factors affecting spatial distribution heterogeneity of different professional cooperatives

**Figure 5:**
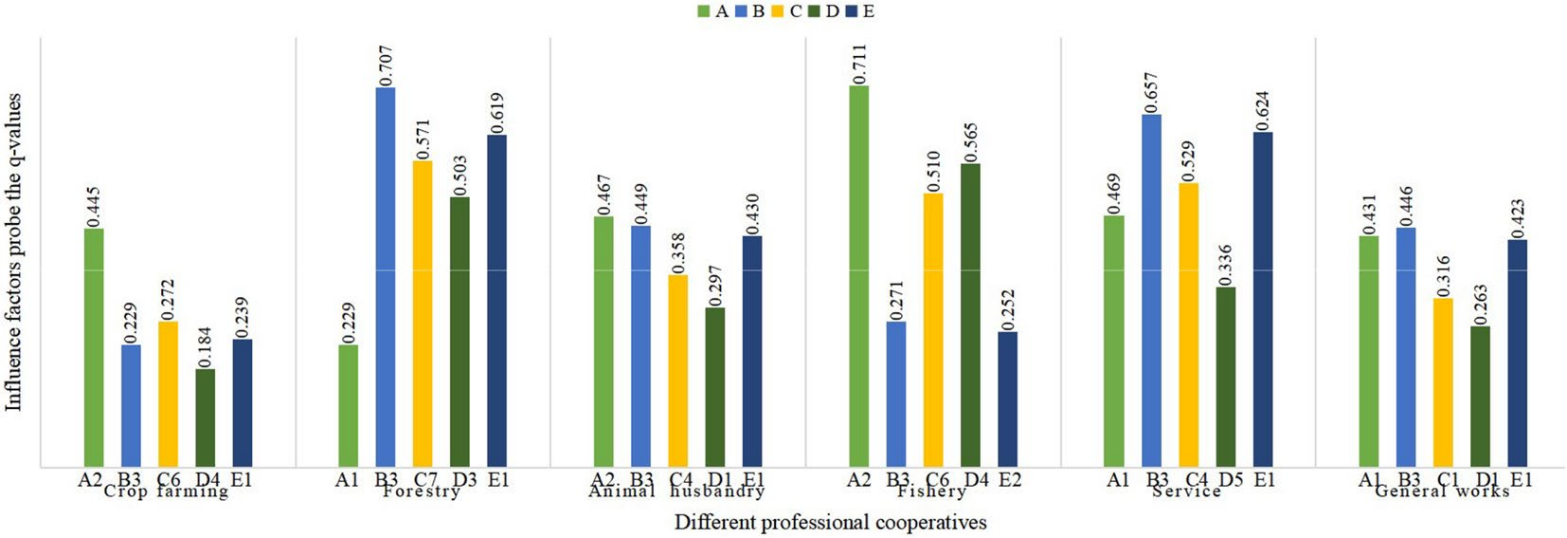
Main influencing factors of spatial distribution of different professional cooperatives (note: "A" represents one of the five dimensions of influencing factors, and "A2" is the influencing factor with the highest q-value within this dimension).

According to the principle of maximum impact, judge the heterogeneity factors of spatial distribution of different professional cooperatives. In the five dimensions: natural ecological environment (A), socio-economic foundation (B), production foundation (C), output capacity (D) and facility foundation (E), select the influencing factor with the largest Q value. They are regarded as the main influencing factors of different professional cooperatives (Fig. 5). The Q-value of influencing factors of farming cooperatives shows that among the five dimensions, the most influential factors are slope (A2), per capita disposable income of rural residents (B3), breeding area (C6), total amount of aquatic products (D4) and road density (E1). The influence value of slope is the largest, while other values are relatively balanced. Forestry cooperatives are affected by the terrain, contrary to planting. Among the five dimensions, the most influential factors are elevation (A1), regional gross product (B4), afforestation area (C5), total output of food crops (D1) and road density (E1), among which road density has the greatest influence. Among the five influencing dimensions of animal husbandry cooperatives, slope (A2), per capita disposable income of rural residents (B3), afforestation area (C5), total output of food crops (D1) and road density (E1) are the most influential ones, among which slope and per capita disposable income of rural residents are the most important. Among them, slope and per capita disposable income of rural residents are the most important factors. The factors that have the greatest influence on fishery cooperatives are aquaculture area (C6) and total aquatic products (D4), with Q values of 0.448 and 0.420, respectively. Road density (E1) is the most influential factor to cooperatives in agriculture, forestry, animal husbandry and fishery services, and the test Q value of the influencing factor reaches 0.622, which is much higher than that of other cooperatives. Among the five dimensions of comprehensive cooperatives, the most influential ones are altitude (A1), regional gross product (B4), afforestation area (C5), fruit yield (D3) and road density (E1).

It can be seen that the topography has an obvious influence on all kinds of professional cooperatives, especially on the cooperatives of planting, animal husbandry, fishery and service industries. Planting cooperatives whose main products are rice, fruits and vegetables are generally distributed in areas with flat terrain and low hills. Regional GDP and per capita disposable income of rural residents play an obvious role in animal husbandry, the service industry and comprehensive cooperatives. Professional cooperatives are affected by agricultural production base and output capacity to varying degrees. For example, the cultivation area and the total amount of aquatic products directly affect the spatial distribution of fishery cooperatives. The cooperatives of agriculture, forestry, animal husbandry and fishery services, mainly agricultural machinery services, plant protection services and tourism services, are greatly affected by afforestation area and fruit output. Finally, the basic conditions of transportation facilities have an obvious influence on forestry, animal husbandry, the service industry and comprehensive cooperatives.

#### Spatial differentiation mechanism of farmer cooperatives

**Figure 6:**
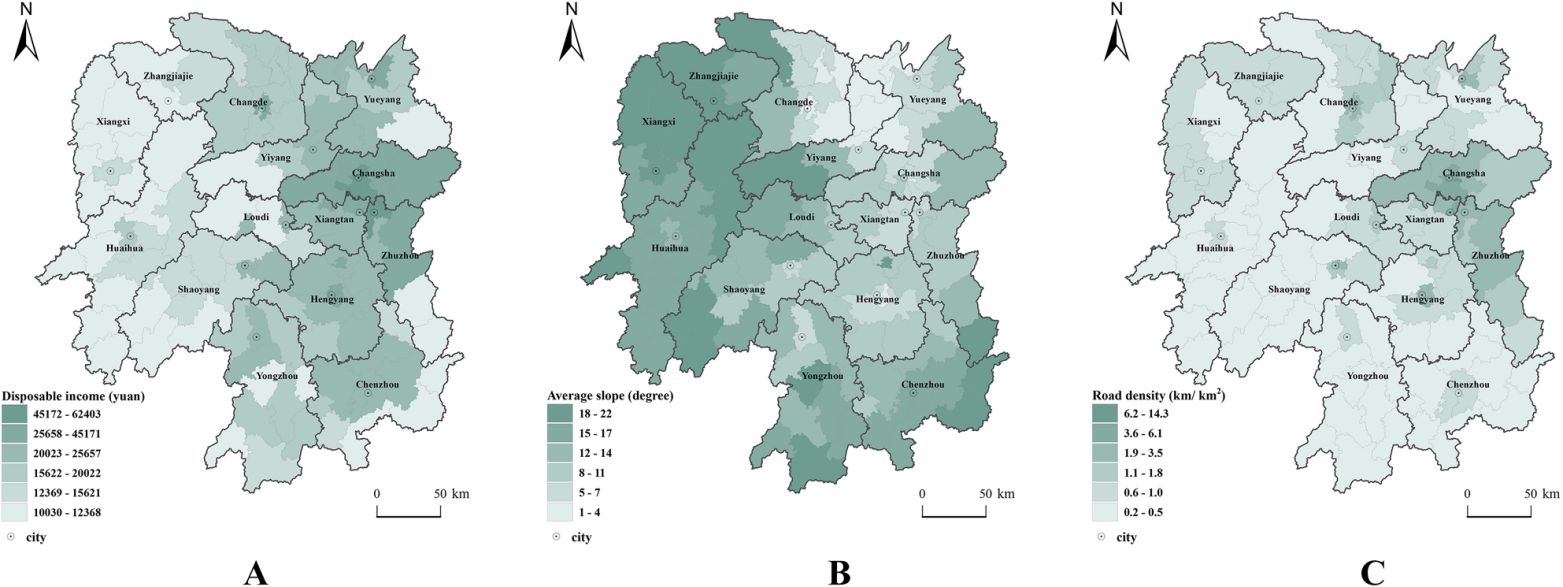
Main influencing factors and distribution points of cooperatives (note: To maximize the distinction between income, slope, and road density, the natural fracture point method in GIS analysis is employed for classification). (**A**) per capita disposable income. (**B**) Slope. (**C**) roading density.

It can be seen that the detection conclusion of this study is consistent with the related research on the influence of the spatial distribution of cooperatives, that is, the development and distribution of cooperatives mainly depend on many reasons such as resource endowment, social and economic development level and market dependence^46^. Through the superposition analysis of the main influencing factors and the distribution points of cooperatives, further verification (Fig. 6) shows that the spatial differentiation mechanism of farmers' cooperatives in Hunan Province is mainly reflected in the following aspects.

Figure 6 was created by the author, who first used the 30m resolution digital elevation data provided by Geospatial Data Cloud (http://www.gscloud.cn). Subsequently, the author used ArcGIS 10.6 software (https://desktop.arcgis.com/zh-cn/quick-start-guides/10.6/) to perform spatial analysis and create color-coded maps.

**Figure 7:**
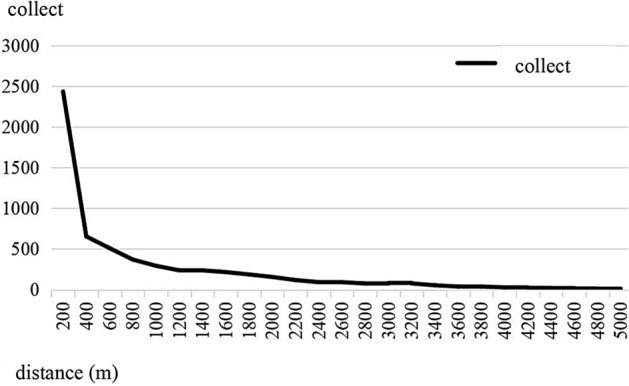
Buffer analysis of cooperative distance road.

1. The natural ecological environment reflects the overall regional environment and resource advantages. It is an important factor affecting the spatial distribution of cooperatives. Cooperatives are mostly distributed in flat areas with little ups and downs. The appropriate altitude and slope can meet the needs of crop planting, which is the basic environment for sustainable agricultural production and the agricultural industrial base formed by farmers' cooperatives. The advantages of the regional environment and resources have especially promoted the development of grain and fishery cooperatives in Dongting Lake Plain.
2. The level of social and economic development reflects the potential market demand, which is the leading factor that affects the spatial distribution of cooperatives. The spatial distribution of cooperatives is polar distribution around the central city of Changsha-Zhuzhou-Xiangtanan agglomeration, which has nothing to do with the social and economic development level of Changsha–Zhuzhou–Xiangtan urban agglomeration. As the region with the highest level of economic development in Hunan Province, Changsha-Xiangtan urban agglomeration's GDP in 2021 is 1923.93 billion yuan, accounting for 41.76% of the province's total, and its per capita disposable income is 48,924 yuan, exceeding the per capita level of China and far higher than that of the other three regions in Hunan Province. Strong market demand is the leading force to promote the development of fruit, vegetable, flower, and seedling cooperatives in Changsha-Zhuzhou-Xiangtan urban agglomeration.
3. Production base and output capacity have some effect on how cooperatives are spread out in space. Compared to the natural ecological environment and the level of social and economic development, the production base and output capacity have a relatively small effect. But the production base and output capacity show the conditions and level of agricultural production in this area. The output capacity of agricultural products can also show how much they are sold commercially. For example, the distribution of cooperatives in Dongting Lake Plain in northern Hunan is concentrated. Nanxian, Huarong, Chengxian, Anxiang, and other districts and counties in this area have gradually developed into important areas for the commercialization of agricultural products due to the increase in agricultural production since the Republic of China. Therefore, the sown area of grain crops, the total irrigated area, and the total output of grain crops still have a certain influence on the spatial distribution of farmers' cooperatives.
4. Accessibility is an important factor influencing the spatial distribution of cooperatives. The infrastructure construction with rural transportation facilities as the main part has a great influence on the development of cooperatives. By the end of 2020, Hunan province has built a highway transportation network with expressways as the backbone, ordinary national and provincial trunk roads as the support and rural roads as the basis, with a total mileage of 241,100 km and the area density of the highway network of 113.85 km/100 square kilometers^47^.
5. This network has a total length of 241,100 km and a surface density of 113.85 km per 100 square kilometers. Among them, the length of rural roads, mainly county roads, township roads and village roads, is 202,000 km, accounting for 83.80%. The continuous improvement of road construction has created favorable traffic conditions for the development of agricultural and rural economy. Through the buffer analysis of the distribution of cooperatives from roads (referring to roads with certain grades), it can be seen that there are 2434 cooperatives within 200 m of roads, accounting for 39.95% of the total, and cooperatives within 1000 m account for 65.18% (Fig. 7).

### Analysis of the influence mechanism of the development of farmer cooperatives

**Figure 8:**
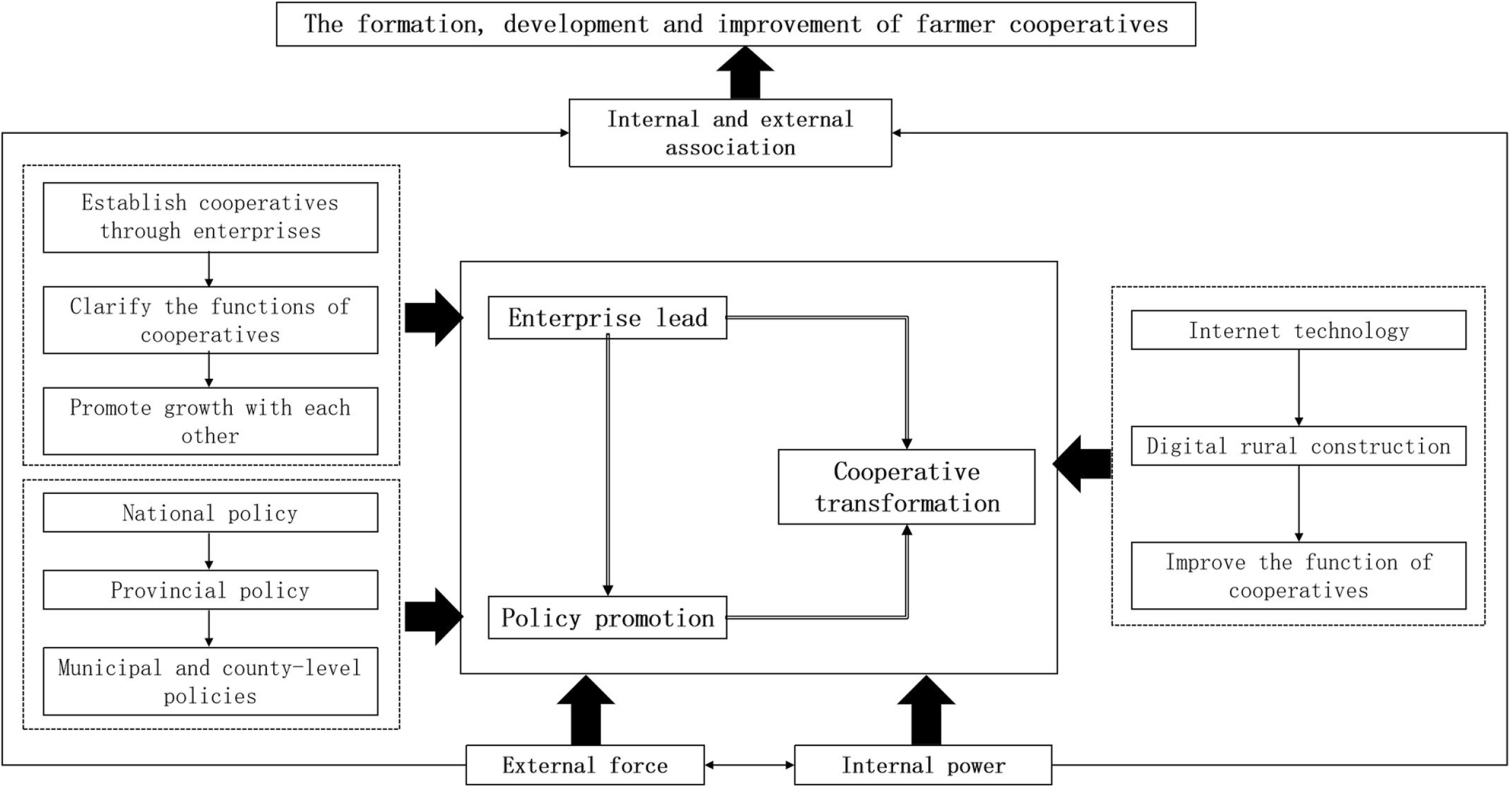
Farmer cooperative development influence mechanism.

The spatial distribution of farmers 'cooperatives is the intuitive reflection and direct embodiment of the regional development differences of cooperatives. However, the spatial distribution of farmers' cooperatives is only one aspect of the development of cooperatives, and the main factors affecting their spatial distribution have been analyzed above. However, there are many other factors affecting the overall development of cooperatives, such as the product characteristics, operation scale of cooperatives, the government's information, technology, training and financial subsidies, which have an impact on the service function, standardization level and high-quality development of cooperatives^48^. The in-depth analysis of other influencing factors for the development of farmers' cooperatives in Hunan Province can be summarized as the three major mechanisms of enterprise leading development, policy promoting development and the transformation and development of cooperatives themselves (Fig. 8). Among them, the first two are external mechanism of action, the latter is internal mechanism of action, internal and external association to promote the comprehensive development of cooperatives.

First, the enterprise-leading development mechanism. Enterprises (mainly referring to the leading enterprises of agricultural industrialization) play a direct leading role in the formation and development of cooperatives. The cooperatives led by the leading enterprises have strong overall operating strength and also have strong strength of joint agriculture. In 2007, the first Hunan province with legal person status—Longping rice grain professional cooperative was established. The cooperative was registered and established by Longping Rice High-tech Co., Ltd., including 1 general office and 11 branches. The main functions of the cooperative include unified procurement of materials, grain purchase, training of farmers, promotion of agricultural machinery services and production technology. Leading enterprises leading the development mode of cooperative is a fundamental change on the cooperative development mechanism, cooperatives as an intermediary, on the one hand, serve the enterprise itself, on the other hand, drive into the club the farmers' income, the farmers, leading enterprises can invest in land and labor in the implementation of incentive compatibility within the governance framework, under the governance framework, both share the remaining control and claim, to achieve the goal of pareto improvement nature^49^.

Second, the role mechanism of policy promotion is obvious. The development of Chinese farmers' cooperatives is greatly affected by the policies, especially the evaluation and generation of demonstration cooperatives, which mainly depends on the promotion of a series of policies. The development and expansion of farmer cooperatives in Hunan province is based on the following two major policies. In 2013, the Opinions of Hunan Provincial People's Government on Accelerating the Development of Farmers' Cooperatives (Xiang Zhengfa [2013] No. 34) proposed to improve the total development of the province's farmers' cooperatives, the number of farmers and the quality of operation in 5 years. In 2014, Hunan Provincial Government implemented two "millions" projects of modern agriculture projects, among which 1000 modern agricultural machinery cooperatives providing full services for rice production were included in the modern agricultural development project of "hundreds of enterprises, thousands of cooperatives and thousands of households", promoting cooperatives to become the most powerful and dynamic new agricultural business entities. In 2019, 11 departments and units, including the Central Agriculture Office and the Ministry of Agriculture and Rural Affairs, jointly issued the Opinions on Improving the Standards of Farmers 'Cooperatives, proposing to increase policy support for the development of farmers' cooperatives by increasing support for financial projects, innovating financial services, and strengthening talent support.

Third, the transformation and development of the cooperative itself mechanism. With the development trend of Internet, digital agriculture and digital rural construction, the function of farmers 'cooperatives is gradually breaking through the limitation of regional scope, which has a great driving role in the transformation and development of farmers' cooperatives. Farmers' cooperatives are an important institution to promote the innovation achievements of agricultural science and technology, and they are also the practitioners and leaders of improving the socialized agricultural socialization service system, mainly taking into account the production, finance, information, sales and other service contents. In addition, with the diffusion and spillover effects of urban innovation, the integrated development of urban and rural industries, and the promotion of the rural revitalization strategy, favorable conditions are provided for the transformation and development of cooperatives themselves.

## Conclusion and discussion

### Conclusion

This study follows the analytical framework of "overall characteristics–spatial pattern–influencing factors–mechanism analysis–development countermeasures" and conducts an in-depth analysis and exploration of farmer cooperatives in Hunan Province. The specific research content includes: firstly, a comprehensive analysis of the overall characteristics of the development of farmer cooperatives in Hunan Province; secondly, revealing the spatial pattern characteristics based on 6328 POI data points of farmer cooperatives and 1954 provincial-level demonstration cooperatives; thirdly, employing the Geographic Detector model to analyze the impact of natural, economic, and social environmental factors on the regional differentiation of farmer cooperatives from the perspectives of prefecture-level cities, agricultural areas, and cooperative professional types. Lastly, conducting a detailed analysis of the development mechanisms of farmer cooperatives from the perspectives of policy promotion, enterprise guidance, and self-transformation.

Study conclusion shows that:

i. The number of farmer cooperatives in Hunan Province exhibits rapid overall growth, with an increasingly strengthened capacity to drive rural industry development. However, there is uneven development among cooperatives of different industrial types, and there is room for improvement in the overall development level of cooperatives. For instance, Table 1 illustrates the relatively small proportion of cooperative demonstration cooperatives in the total number of cooperatives.
ii. The overall spatial distribution of farmer cooperatives in Hunan Province exhibits a pattern characterized by "one core, one ring, and multiple points." The central areas around Changsha, Xiangtan, and Zhuzhou cities serve as the core, presenting a spatial form of "large agglomeration and radial distribution." The spatial aggregation of cooperatives is significant.
iii. Firstly, Table 2 reflects significant differences in the number of cooperatives of different industry types in the POI data. Secondly, six major industry types, namely planting, forestry, animal husbandry, fisheries, agriculture-forestry-animal husbandry-fishery services, and comprehensive, exhibit distinct spatial distribution characteristics. They respectively present patterns such as anti-"L" shaped chain, dual-core, central with surrounding, polar, radial, and cluster.
iv. Based on the natural ecological environment, social and economic foundation, production, output capacity, facilities, five dimensions selected 24 influence factor detection analysis, the influence of the factors is higher, general, weak three levels, the results of the cooperative spatial distribution mainly by resources endowment, social and economic development level and the influence of market dependence, etc. Figure 6 illustrates the most significant factors impacting the spatial distribution of different professional cooperatives.
v. There is heterogeneity in factors influencing the spatial distribution of 6 major industrial types, mainly influenced by different degrees of agricultural production base and output capacity. But on the whole, the influence of terrain, gross regional product, per capita disposable income of rural residents, transportation infrastructure is more obvious.
vi. Further analyze the influence mechanism of cooperative development from the perspectives of enterprises, policies and cooperative ontology. It is proposed that enterprises play a direct leading role in the formation and development of cooperatives, the policy promotion mechanism is obvious, and the development trends such as Internet, digital agriculture and digital rural construction have a great driving role in the transformation and development of farmers' cooperatives.

### Suggestions for the innovation and development of cooperatives

Based on the analysis of the above spatial distribution and influencing factors, as well as the theoretical exploration of the innovative development mechanism of farmers 'cooperatives, this paper puts forward the following suggestions and thoughts on the innovation and development of farmer cooperatives in Hunan Province.

1. Improving Cooperative Development Policies in a Hierarchical and Categorized Manner. Encourage and advance policies supporting the development of farmers' cooperatives are predominantly at the national and provincial levels, with encouragement policies at the city and county levels lagging behind or being relatively insufficient, given local realities. Therefore, it is imperative to formulate policies that promote cooperative development in a tiered manner. National and provincial-level policies should focus on supporting the development and growth of farmers' cooperatives through agricultural production development funds. At the city and county levels, policies should be tailored to regional development needs, enhancing the technological application and production management capabilities of cooperatives through effective training and organizational initiatives. This tiered approach aims to propel the development of farmers' cooperatives. Guide innovative cooperative development through categorization. Currently, policy support for farmers' cooperatives tends to favor well-established enterprise-led cooperatives, including agricultural technology services, agriculture-related projects, and agricultural machinery subsidies, among other services and reward measures. The categorized guidance development mechanism should first overcome the tendency to excessively focus on "supporting the best and the strong." It should encompass policies supportive of small and medium-sized as well as initial-stage farmers' cooperatives, formulating support policies aligned with different stages of development. This approach aims to strengthen the precision and functionality of policy guidance. Additionally, attention should be directed towards strengthening the classification and guidance of policies for cooperatives of different industrial types. The "Hunan Province Farmers' Cooperatives Provincial Demonstration Society Creation Method" emphasizes key support for the development of food, livestock, poultry, vegetables, tea, oil, Chinese medicinal materials, aquatic products, and other characteristic industries of farmers' cooperatives. This approach should be further integrated with different industry-type cooperatives in organizing production, aligning with the market, and enhancing promotional services, thereby refining policies and measures to meet specific industry demands.
2. Subregional optimization measures to promote the development of cooperatives. In consideration of the spatial distribution characteristics of farmers' cooperatives in Hunan Province, the Changsha-Zhuzhou-Xiangtan urban agglomeration, characterized by better economic and social development conditions, serves as the primary distribution hub. Other distribution areas predominantly include main agricultural product-producing regions (counties and cities). Optimization measures for zoning should focus on differentiated policies. Firstly, enhance encouraging policies for farmers' cooperatives in regions with underdeveloped economic and social conditions. Secondly, for areas lacking distinct advantages in agricultural production, emphasize improvements to basic agricultural production conditions, stimulate regional agricultural product circulation, and reinforce the development of agricultural industry projects. Concerning specific spatial considerations, alignment with the agricultural spatial planning of "four areas and one base" in Hunan Province is crucial. These include the Changsha-Zhuzhou Urban Agricultural area, Pinghu Agricultural area around Dongting Lake, Wuling Xuefeng Nanling Luoxiao Mountain Agricultural Area, and the hilly water-saving agricultural area in southern Hunan. This alignment aims to establish a national green agricultural products base grounded in the four agricultural areas. Elevating the high-quality development level of Xiangtan's 3 + 5 urban cooperatives and extending support to the Xiangxi region and the rural revitalization demonstration counties (cities, areas) in southern Hunan is paramount. This strategic approach seeks to foster a more balanced layout of farmers' cooperatives in the province, ensuring greater equilibrium in agricultural socialization and organizational levels, thereby advancing the establishment of a new pattern of modern agricultural development in Hunan Province.
3. Further improve the development model of enterprises leading cooperatives. To achieve this, several key measures re recommended. Firstly, enhancing the industrial, supply, and value chains of enterprises can significantly boost their production capacity, market responsiveness, and innovation capabilities. This, in turn, will strengthen the overall ability of enterprises to lead the development of both cooperatives and farmers. Secondly, there should be a focus on optimizing the structural creation model of cooperatives and farmers, guided by enterprises. Improving the modern agricultural management system, solidifying the foundation of agricultural industrialization, and overcoming constraints related to land, labor force, capital, and market are crucial steps. These efforts aim to enhance the efficiency of rural resource allocation and the scale efficiency of agricultural economic development. Thirdly, it is imperative to reinforce the internal supervision mechanism. This involves improving the standardization of financial fund utilization and the clarity of financial accounting within cooperatives under enterprise guidance. Establishing a long-term mechanism for the implementation, management, protection, and evaluation of cooperatives, supported by policy initiatives, is essential for sustained success.
4. Improve the innovation and development ability of farmers' cooperatives. The innovation and development ability of farmers 'professional cooperatives has a significant role in the innovation of agricultural management subjects, the improvement of farmers' organization degree and the ability to participate in market competition^39^. Farmers' cooperatives are an important component of the rural innovation system. Strengthening the promotion function of cooperative scientific and technological innovation, optimizing the social service innovation of cooperatives and promoting the innovation of cooperative organization will be conducive to improving the overall function of the rural innovation system. Optimize cooperative service innovation, should consider the agricultural production data purchase, seed introduction and technology promotion such as prenatal services, concentrated seedling seedlings, machine sowing machine mechanized services, fertilizer, irrigation, drainage, epidemic prevention and control in production services, as well as agricultural products processing, storage and transportation, product quality inspection and other postpartum services^50^.

The foundation of the stability of farmer cooperatives lies in the regional dependence, which is the driving force mechanism of farmer cooperative innovation^29^. Through the expansion of living space, the utilization of superior resources and the innovation of cooperation mode can promote the growth and evolution of cooperatives^11^. Cooperatives connecting enterprises, family farms, farmers and other cooperatives also have various different modes of innovation in the profit distribution mechanism, operation mechanism and participation mechanism.

### Further summary and discussion

This study, based on the classification of farmer cooperative types, utilizes POI data points of farmer cooperatives to measure the spatial pattern of farmer cooperatives in Hunan Province at the county scale. By employing GIS spatial analysis and geographic detector methods, the study scientifically summarizes the spatial distribution characteristics of different types of cooperatives and their spatial agglomeration features. An indicator system is constructed to analyze in-depth the influencing factors of spatial differentiation, and further qualitative analysis is conducted on other influencing mechanisms of cooperative development. The study achieves a comprehensive application of quantitative and qualitative analyses, providing a scientific assessment of the spatial pattern and development mechanisms of farmer cooperatives in Hunan Province.

However, some data limitations pose challenges to the empirical quantitative analysis in this study. According to statistics, the number of farmer cooperatives in Hunan Province reached 93,500 in 2020. Nevertheless, after filtering through the POI data obtained using the programming language tool Python 3.8, only 6328 actual valid data points were obtained. While the obtained demonstration cooperative data is relatively comprehensive, there are still some data points that could not be accessed due to lack of public availability, potentially limiting the comprehensive interpretation of the spatial pattern of farmer cooperatives across the entire province.

Regarding the selection of influencing factors, the study focused on quantitative research using indicators at the county level, lacking specific indicators for the development of individual cooperatives. This limitation makes it challenging to fully explain the influencing mechanisms of spatial differentiation of cooperatives. In future research, the focus will shift to the micro-level analysis of farmer cooperatives, incorporating specialized data collection through surveys and continually refining the study of cooperatives by combining qualitative indicators with quantification.

## Data Availability

The research results reported in this article are based on the data sets generated and/or analyzed in this study. Due to the fact that these data sets contain surveying and mapping geographic information and data from China, in accordance with the requirements of relevant national laws and regulations, we are unable to make these data sets publicly available. However, in support of the spirit of scientific research sharing, we are willing to provide these data sets to interested researchers under reasonable requests. Researchers who wish to obtain the data sets can contact the corresponding author via email. We will determine whether to provide the data sets and the specific conditions of the provision based on the nature of the request and the sensitivity of the data.

## References

[1] The law of the People's Republic of China on specialized farmer cooperatives[EB/OL]. http://www.npc.gov.cn/zgrdw/npc/lfzt/rlyw/2017-06/26/content_2024367.htm (2017).

[2] Mao YP, Li GB, Wang Y (2016). Study on interaction mechanism between the rural cooperative economy and rural space: From actor-network theory in Suzhou. Urban Dev. Stud..

[3] Wang ZB, Wang XM (2019). Analysis of the spatial and temporal evolution and influencing factors of farmers' professional cooperatives in Beijing-Tianjin-Hebei. Acta Ecol. Sin..

[4] Reply to recommendation No. 1004 of the fourth session of the Thirteenth National People's Congres [EB/OL] (2021). http://www.moa.gov.cn/govpublic/NCJJTZ/202106/t20210615_6369582.htm.

[5] Wang JP, Ren DP (2016). "Model" standard: Research on the selection criteria of model cooperatives of farmers' professional cooperatives. J. China Agric. Univ. (Soc. Sci. Edition).

[6] Summary of the reply to proposal No. 2539 of the fourth session of the 13th national committee of the CPPCC [EB/OL] (2021). http://www.moa.gov.cn/govpublic/NCJJTZ/202107/t20210722_6372536.htm.

[7] Circular of the ministry of agriculture and rural affairs of the People's Republic of China on the implementation of the action to improve new agricultural business entities [EB/OL] (2022). http://www.hzjjs.moa.gov.cn/gzdt/202203/t20220325_6394044.htm.

[8] Konstantina R, Evgenia A, George T, Konstantinos K (2021). Democratic administration and commitment of members of agricultural cooperatives: A case study from a prefecture in Greece. Businesses.

[9] Davron N, Ilkhom S, Insa T (2021). Ordered to volunteer? Institutional compatibility assessment of establishing agricultural cooperatives in Uzbekistan. Land Use Policy.

[10] Yu L, Huang W (2020). Non-economic societal impact or economic revenue? A performance and efficiency analysis of farmer cooperatives in China. J. Rural. Stud..

[11] Gava O, Ardakani Z, Delalić A, Azzi N, Bartolini F (2021). Agricultural cooperatives contributing to the alleviation of rural poverty. The case of Konjic (Bosnia and Herzegovina). J. Rural Stud..

[12] Neves MDR, Silva FD, Freitas CO (2021). The role of cooperatives in Brazilian agricultural production. Agriculture.

[13] Hoken H, Su Q (2018). Measuring the effect of agricultural cooperatives on household income: Case study of a rice-producing cooperative in China. Agribusiness.

[14] Ahado S, Hejkrlík J, Enkhtur A (2021). Does cooperative membership impact the yield and efficiency of smallholder farmers? Evidence from potato farmers in Mongolia China. Agric. Econ. Rev..

[15] Liu TSH, Wu G (2022). Does agricultural cooperative membership help reduce the overuse of chemical fertilizers and pesticides? Evidence from rural China. Environ. Sci. Pollut. Res. Int..

[16] Dong Y, Mu Y, Abler D (2019). Do farmer professional cooperatives improve technical efficiency and income? Evidence from small vegetable farms in China. J. Agric. Appl. Econ..

[17] Tran GTH, Teruaki N, Yosuke C (2023). The impact of cooperative participation on income: The case of vegetable production in Vietnam. J. Agribus. Dev. Emerg. Econ..

[18] Fetia WW, Tuti K, Iwan S (2023). The effect of performance on the sustainability of coffee farmers’ cooperatives in the industrial revolution 4.0 in west Java Indonesia. Sustainability.

[19] Grashuis J (2019). Agricultural firm survival: The case of farmer cooperatives in the United States. Agribusiness.

[20] Zhang Y, Huang ZH (2014). Identifying risks inherent in farmer cooperatives in China. China Agric. Econ. Rev..

[21] Gerald M (2022). Evaluating changes in credit rating quality of US farmer cooperatives. J. Co-oper. Organ. Manag..

[22] Yu L, Nilsson J (2018). Societal capital and the financing performance of farmer cooperatives in Fujian Province China. Agribusiness.

[23] Liu JY, Liang JR, Zhang SCH (2022). The spationtemporal distribution and evolution characteristics of farmer professional cooperatives in hilly and mountainous areas: A case study in Chongqing. Chin. J. Agric. Resour. Region. Plann..

[24] Ma YI (2019). Farmland shareholding cooperatives’ fixed contracts are better than shared contracts. Issues Agric. Econ..

[25] Kang MZ, Wang XJ, Wang HY (2020). Digital cooperatives: Agricultural intelligent system integrating production and marke. Res. Agric. Modern..

[26] Yun S, Jinmin W, Luyao W (2022). How do cooperatives alleviate poverty of farmers? Evidence from rural China. Land.

[27] Kumar D, Peshin R, Kumar P (2022). Farmers perceptions of dairy cooperatives: Evidence from subtropics of Jammu. J. Community Mob. Sustain. Dev..

[28] Huma N (2022). Impact of cooperative membership on production efficiency of smallholder goat farmers in Nepal. Ann. Public Cooper. Econ..

[29] Molla A, Azanaw A, Assefa D (2024). Determinants of dairy cooperative membership among smallholder farmers in North-Western Ethiopia. Cogent Food Agric..

[30] Zhao, K., Cao, J.J., Zhang Y., et al. Spatial differentiation characteristics of new agricultural business entities and their innovative development mechanism: Take Shandong Province as an example.* China’s Agric. Resour. Region.* **1**, 1–15 (2024).

[31] Filippi M, Triboulet P, Chantelot S (2015). The spatial distribution of French agricultural cooperatives: An exploratory spatial data analysis. Eur. Plan. Stud..

[32] Xiong Y, Huang LH, Zou F (2021). Based on the multi-functional spatial differentiation characteristics and types of rural areas: Take Hunan Province as an example. Econ. Geogr..

[33] Chen HQ, Zeng FSH (2022). Measurement of development level of agricultural and rural modernization in Hunan Province. Econ. Geogr..

[34] Li, J. Z. H. Exploration on the management situation of farmer professional cooperative economic organization: Take 48 farmer professional cooperative economic organizations in Hunan province as an example. *Heilongjiang Anim. Husband. Vet. Med.* **18**, 33–37 (2016).

[35] Statistical yearbook-Hunan provincial people's government portal website [EB/OL]. https://www.hunan.gov.cn.

[36] Notice of the ministry of agriculture and rural affairs on the application of national farmers' cooperatives in 2022[EB/OL] (2022). https://www.moa.gov.cn/xw/bmdt/202205/t20220518_6399632.htm.

[37] Zhu Z, Yamg H, Hu YM (2021). Evaluation of village development potential and village classification by multi-source data. J. Agric. Resour. Environ..

[38] Xue B, Li JZ, Xiao X (2019). Overview of man-land relationship research based on POI data: Theory, method and applocation. Geogr. Geo-Inf. Sci..

[39] Chen WQ, Dai YQ, Geng YW (2020). Village classification method based on POI data and gravity model. Trans. Chin. Soc. Agric. Mach..

[40] Wang JF, Xu CHD (2017). Geodetector: Principle and prospective. Acta Geogr. Sci..

[41] Luo L, Qiao D, Zhang R (2022). Research on the influence of education of farmers’ cooperatives on the adoption of green prevention and control technologies by members: Evidence from rural China. Int. J. Environ. Res. Public Health.

[42] Deng H, Huang J, Xu Z (2010). Policy support and emerging farmer professional cooperatives in rural China. China Econ. Rev..

[43] Zhou N (2022). Research on urban spatial structure based on the dual constraints of geographic environment and POI big data. J. King Saud Univ. Sci..

[44] Min RUN (2023). Study on the spatial distribution of new agricultural operators in Tengzhou City based on POI data. Mod. Agric..

[45] Zhu KY, Wei L (2023). Study on the influencing factors of the cultivation of new agricultural business subjects in Henan Province. China's Agric. Resour. Region..

[46] Liu SH, Guo Y, Tian OUN (2014). Distribution characteristics and reason of farmers' specialized cooperatives: taking example for first batch of farmers' professional cooperative model. Sci. Geogr. Sin..

[47] Highway Network Layout Planning of Hunan Province (2021–2050)[EB/OL] (2022).http://jtt.hunan.gov.cn/jtt/xxgk/ghjh/202201/t20220107_21600715.html.

[48] Liao XJ, Deng HSH, Shen GY (2021). Research on the high-quality development mechanism of farmers' cooperatives. J. Nanjing Agric. Univ. (Social Science Edition).

[49] Deng HT, Zhao Y, Yang Y (2020). From cooperative to cooperative association: the economic logic of contract selection of leading enterprises and farmers under market expansion: Take a leading enterprise and land cooperative in Taigu County, Shanxi Province as an example. J. Manage World.

[50] Jiang ChY (2018). Research on the development relationship between leading enterprises and farmer cooperatives and family farms. Soc. Sci. Front..

